# *Clostridioides difficile* infection among patients with type 2 diabetes mellitus

**DOI:** 10.1007/s11739-025-04168-y

**Published:** 2025-11-03

**Authors:** Sameer Kassem, Mohamed Atamna, Nili Stein, Adnan Zaina

**Affiliations:** 1 Faculty of Medicine, Technion-Institute of Technology, Haifa, Israel; 2 Department of Internal Medicine, Carmel Medical Center, Ruth and Bruce Rappaport, Haifa, Israel; 3 Department of Community Medicine and Epidemiology, Statistical Unit, Lady Davis Carmel Medical Center, Ruth and Bruce Rappaport Faculty of Medicine, Technion-Institute of Technology, Haifa, Israel; 4 Department of Translational Epidemiology Unit and Research Authority, Lady Davis Carmel Medical Center, Ruth and Bruce Rappaport Faculty of Medicine, Technion-Institute of Technology, Haifa, Israel; 5 Department of Endocrinology and Metabolism, Clalit Medical Health Care Services, Haifa, Israel; 6 Zvulon Medical Center and Bar-Ilan University, The Azrieli Faculty of Medicine, Safed, Israel

**Keywords:** *Clostridioides difficile* infection, Diabetes mellitus, Comorbidities, Mortality

## Abstract

*Clostridioides difficile* infection (CDI) poses a significant healthcare burden. Patients with diabetes mellitus (DM) are at increased risk of poor outcomes. This study aims to compare CDI outcomes, including mortality, in patients with and without DM. Retrospective data between (2014–2024) from Clalit Health Services (CHS) electronic database were analyzed. Among 2319 patients with confirmed CDI, 1005 had DM, and 1314 did not. DM patients were significantly older (74.5 ± 12.0 vs. 68.6 ± 20.4 years; *p* < 0.001), more likely to be male (p = 0.029), and disproportionately represented in lower socioeconomic status (SES) groups (30.1% vs. 22.4%; *p* < 0.001). Additionally, patients with DM had a significantly higher prevalence of comorbidities, with a median Charlson comorbidity Index (CCI) of 7.5 compared to 4.9 (p < 0.001), along with higher serum creatinine levels and lower albumin levels (p < 0.001). All-cause mortality was significantly higher in the DM group (p < 0.001). Deceased patients were notably older (p < 0.001), more likely male, and had lower SES (27% vs. 25.1%, p = 0.028). Patients with DM and CDI exhibited different comorbidities compared to those without diabetes. DM, advanced age, low SES, and male gender are linked to poorer outcomes, including mortality, among patients with CDI. These findings underscore the need for intensive management in patients with diabetes and CDI.

## Introduction

*Clostridioides difficile* infection (CDI) represents a significant healthcare-associated disease characterized by a marked increase in incidence and pathogen virulence in recent years, resulting in substantial morbidity and mortality [1]. CDI is a leading cause of nosocomial outbreaks in hospitals and healthcare settings and has emerged as a critical concern for healthcare systems worldwide [2].

The clinical presentation varies from uncomplicated diarrhea to life-threatening clinical syndromes, including pseudomembranous colitis, which could be complicated by toxic megacolon, septic shock, and intestinal perforation [3].

Different risk factors for developing CDI have been described, mainly exposure to antibiotic treatment, as well as hospitalization, increase in the length of hospitalization, admission to intensive care unit (ICU), medical device use, proton pump inhibitors (PPI) use, and immune suppression/dysfunction; all of which contributes to microbiota alterations in the gastrointestinal tract which plays a significant role in CDI development [4, 5].

Diabetes Mellitus (DM) is recognized as a worldwide pandemic resulting in high rates of hospitalization and medical care utilization [6, 7]. It has been demonstrated that patients with DM are more prone to infections and infection-related complications than non-DM patients [8]. Notably, DM is associated with immune dysfunction, which includes adverse effects on innate immunity and impaired functions of white blood cells, thereby increasing the risk of infections [9]. Furthermore, the composition of intestinal microbiota in DM is altered when compared to non-diabetics; for example, there is a decrease in Firmicutes and an increase in Bacteroidetes and Proteobacteria, which play an essential role in developing CDI in DM patients [10].

DM is a significant risk factor for developing CDI [11]. It is associated with higher recurrence rates of CDI and is considered a risk factor for treatment failure in CDI [12]. Infections display a significant challenge for patients with DM. However, whether DM is an independent risk factor for CDI remains to be investigated. Scanty published data comparing CDI among patients with DM and those without DM; therefore, our current research evaluates the clinical outcomes of patients with CDI during hospitalization in both groups (diabetic and non-diabetic), including baseline characteristics associated comorbidities, differences in primary diagnosis at hospitalization, 90 day mortality and, factors related to the outcome of interest (e.g., mortality within 90 days) by using different variables including diabetes itself.

## Methods and materials

Retrospective data were collected from Clalit Health Services (CHS) between 2014 and 2024 using an electronic database. Patients aged 18 years or older with a confirmed diagnosis of CDI, verified by a positive ELISA test for *Clostridioides difficile* toxin and confirmed by PCR tests for toxins A and B, were included in the study and considered the index day (ID). The primary outcome of our current research is to determine the relationship between mortality among patients with and without diabetes diagnosed with CDI. Demographic characteristics, including age, gender, ethnicity, socioeconomic status, and associated comorbidities (such as heart failure, chronic obstructive pulmonary disease, malignancy, and inflammatory bowel disease), as well as functional status, the Charlson Comorbidity Index, and frailty score, were obtained. The primary diagnosis at the time of hospitalization was reported. Laboratory tests for albumin, creatinine, and WBC were collected.

### Statistical methods

Continuous variables were summarized with mean ± standard deviation and categorical variables were presented as numbers and proportions.

Comparisons of baseline characteristics between patients with DM and non-DM were performed using the Chi-Square test for categorical variables and the Independent t–test, or Mann–Whitney, as appropriate, for the continuous variables. Time to mortality (Follow-up: 90 days) was estimated using Kaplan-Meir curves, and a log-rank test was used to compare the difference between diabetic and non-diabetic patients.

Cox proportional hazards regression was used to evaluate the relationship between diabetes and 90 day mortality among patients diagnosed with CDI, adjusting for demographic and clinical factors using backward elimination methods.

Statistical analysis was performed using IBM Statistics vs. 28. P < 0.05 was considered statistically significant. The Institutional Review Board (Helsinki Committee) at Carmel Hospital, along with the ethics committee, approved the study.

## Results

### Baseline demographic and clinical characteristics

A total of 2319 patients diagnosed with CDI were included in this study, comprising 1005 patients with diabetes and 1314 without diabetes. The baseline characteristics of these patients are summarized in Table 1.

**Table 1:** Patients baseline demographics and clinical characteristics

Patient characteristics	Total n = 2319	No diabetes n = 1314	Diabetes n = 1005	P Value
Age means (SD)	71.2 ± 17.6	68.6 ± 20.4	74.5 ± 12.0	< 0.001
Male n (%)	1050 (45.3)	569 (43.3)	481 (47.9)	0.029
SES n (%)				< 0.001
Low	597 (25.7)	294 (22.4)	303 (30.1)	
Middle	1417 (61.1)	813 (61.9)	604 (60.1)	
High	292 (12.6)	200 (15.2)	92 (9.2)	
Missing	13 (0.6)	7 (0.5)	6 (0.6)	
Ethnicity (Jews) n (%)	2066 (89.1)	1204 (91.6)	862 (85.8)	< 0.001
Hypertension n (%)	1648 (71.1)	751 (57.2)	897 (89.3)	< 0.001
Hyperlipidemia n (%)	1774 (76.5)	854 (65.0)	920 (91.5)	< 0.001
IHD n (%)	907 (39.1)	367 (27.9)	540 (53.7)	< 0.001
COPD n (%)	366 (15.8)	177 (13.5)	189 (18.8)	< 0.001
Heart failure n (%)	646 (27.9)	249 (18.9)	397 (39.5)	< 0.001
CVA n (%)	660 (28.5)	285 (21.7)	375 (37.3)	< 0.001
CRF n (%)	909 (39.2)	359 (27.3)	550 (54.7)	< 0.001
Dialysis n (%)	147 (6.3)	47 (3.6)	100 (10.0)	< 0.001
Disability n (%)	813 (35.1)	402 (30.6)	411 (40.9)	< 0.001
Charlson score median	6.0 ± 3.1	4.9 ± 3.0	7.5 ± 2.6	< 0.001
(IQR)	6 (4; 8)	5 (3, 7)	7 (6; 9)	
WBC (n)	N = 2311	N = 1309	N = 1002	0.098
Mean ± SD	12.4 ± 9.6	12.1 ± 8.7	12.8 ± 10.6	
(IQR)	10.6 (7.1; 15.4)	10.4 (7.0; 15.3)	10.8 (7.3; 15.7)	
Creatinine mg/dL (n)	N = 2310	N = 1309	N = 1001	< 0.001
Median (IQR)	0.96 (0.64; 1.67)	0.8 (0.6; 1.27)	1.2 (0.78; 2.2)	
Albumin (n)	N = 2249	N = 1266	N = 983	< 0.001
Mean ± SD	2.8 ± 0.62	2.9 ± 0.65	2.7 ± 0.59	
Median (IQR)	2.8 (2.4; 3.3)	2.9 (2.4;3.3)	2.7 (2.3; 3.2)	

*SES* Socioeconomic status, *IHD* Ischemic heart disease, *COPD* Chronic obstructive pulmonary disease, *CVA* Cerebrovascular accident, *CRF* Chronic renal failure, *IQR* Interquartile range

Patients with diabetes were significantly older, with a mean age of 74.5 ± 12.0 years compared to 68.6 ± 20.4 years for non-diabetic patients (p < 0.001). The proportion of male patients was slightly higher in the diabetic group (47.9% vs. 43.3%, p = 0.029). Socioeconomic status (SES) also varied, with a larger percentage of patients with diabetes belonging to lower SES (30.1% vs. 22.4%, p < 0.001). In contrast, non-diabetic patients were more likely to be categorized as high SES (15.2% vs. 9.2%). The ethnic distribution indicated that a greater proportion of non-diabetic patients were of Jewish descent (91.6% vs. 85.8%, p < 0.001).

Regarding comorbidities, patients with DM showed a significantly higher prevalence of conditions such as hypertension (89.3% vs. 57.2%), hyperlipidemia (91.5% vs. 65.0%), ischemic heart disease (53.7% vs. 27.9%), COPD (18.8% vs. 13.5%), heart failure (39.5% vs. 18.9%), cerebrovascular accidents (37.3% vs. 21.7%), chronic renal failure (54.7% vs. 27.3%), and the need for dialysis (10.0% vs. 3.6%) (all p < 0.001). Additionally, disability was more common among diabetic patients (40.9% vs. 30.6%), and they exhibited a significantly higher Charlson Comorbidity Index (median 7.5, IQR: 6–9) compared to non-diabetic patients (median 4.9, IQR: 3–7) (p < 0.001), indicating a greater overall disease burden.

In terms of laboratory findings, there was a non-significant difference in white blood cell count between diabetic and non-diabetic patients (12.4 ± 9.6 vs. 12.1 ± 8.7, p = 0.098). Additionally, patients with diabetes exhibited significantly higher creatinine levels (1.48 ± 1.48 mg/dL vs. 1.2 ± 1.2 mg/dL, p < 0.001), indicating worse kidney function. Diabetic patients also had lower serum albumin levels (2.8 ± 0.62 g/dL vs. 2.9 ± 0.65 g/dL, p < 0.001), suggesting poorer nutritional or inflammatory status. It is worth noting that laboratory tests were performed 6–24 h before CDI diagnosis.

### Main diagnosis at admission

Table 2 compares the primary diagnoses at hospital admission between patients with diabetes and those without diabetes, all of whom were diagnosed with CDI infection. Among the 2319 patients diagnosed with CDI, cardiovascular diseases were more frequently the primary diagnosis in diabetic patients (10.8% vs. 6.0%), as were bloodstream infections (11.1% vs. 8.8%), pulmonary non-infectious diseases (6.8% vs. 5.1%), and inflammatory and metabolic diseases (6.0% vs. 3.1%). Genitourinary infections were slightly more prevalent among diabetic patients (4.9% vs. 4.2%). In contrast, gastrointestinal infections were more often the primary diagnosis in non-diabetic patients (8.5% vs. 4.2%), along with gastrointestinal non-infectious diseases (6.3% vs. 3.6%) and Clostridium difficile infection itself (24.8% vs. 19.9%). Respiratory tract infections, malignancies, and other musculoskeletal and central nervous system infections were similarly distributed between the two groups. Musculoskeletal diseases and surgical conditions accounted for equal proportions of primary diagnoses in both groups (2.5% and 4.0%, respectively), while other conditions made up about 10% of admissions.

**Table 2:** Main diagnosis at admission

Group of patients Comorbidities	Non-DM n %	DM n%	Total n %
Cardiovascular Diseases	77 (6%)	105 (10.8%)	182 (8.1%)
Respiratory tract infections	87 (6.8%)	67 (6.9%)	154 (6.8%)
Gastrointestinal infections	108 (8.5%)	41 (4.2%)	149 (6.6%)
Genitourinary infections	54 (4.2%)	48 (4.9%)	102 (4.5%)
Bloodstream infections	112 (8.8%)	108 (11.1%)	220 (9.8%)
Clostridium infection	316 (24.8%)	194 (19.9%)	510 (22.7%)
Other infections (e.g. Musculoskeletal, CNS)	26 (2.1%)	28 (2.9%)	54 (2.4%)
Malignancy	94 (7.4%)	62 (6.4%)	156 (6.9%)
Inflammatory and metabolic	40 (3.1%)	60 (6%)	100 (4.4%)
Musculoskeletal	32 (2.5%)	24 (2.5%)	56 (2.5%)
Surgery	50 (3.9%)	39 (4%)	89 (4%)
Pulmonary non-infectious	65 (5.1%)	66 (6.8%)	131 (5.8%)
Gastrointestinal non-infectious	81 (6.3%)	35 (3.6%)	116 (5.2%)
Other	134 (10.5%)	97 (10%)	231 (10.3%)

### All-cause mortality

**Fig. 1:**
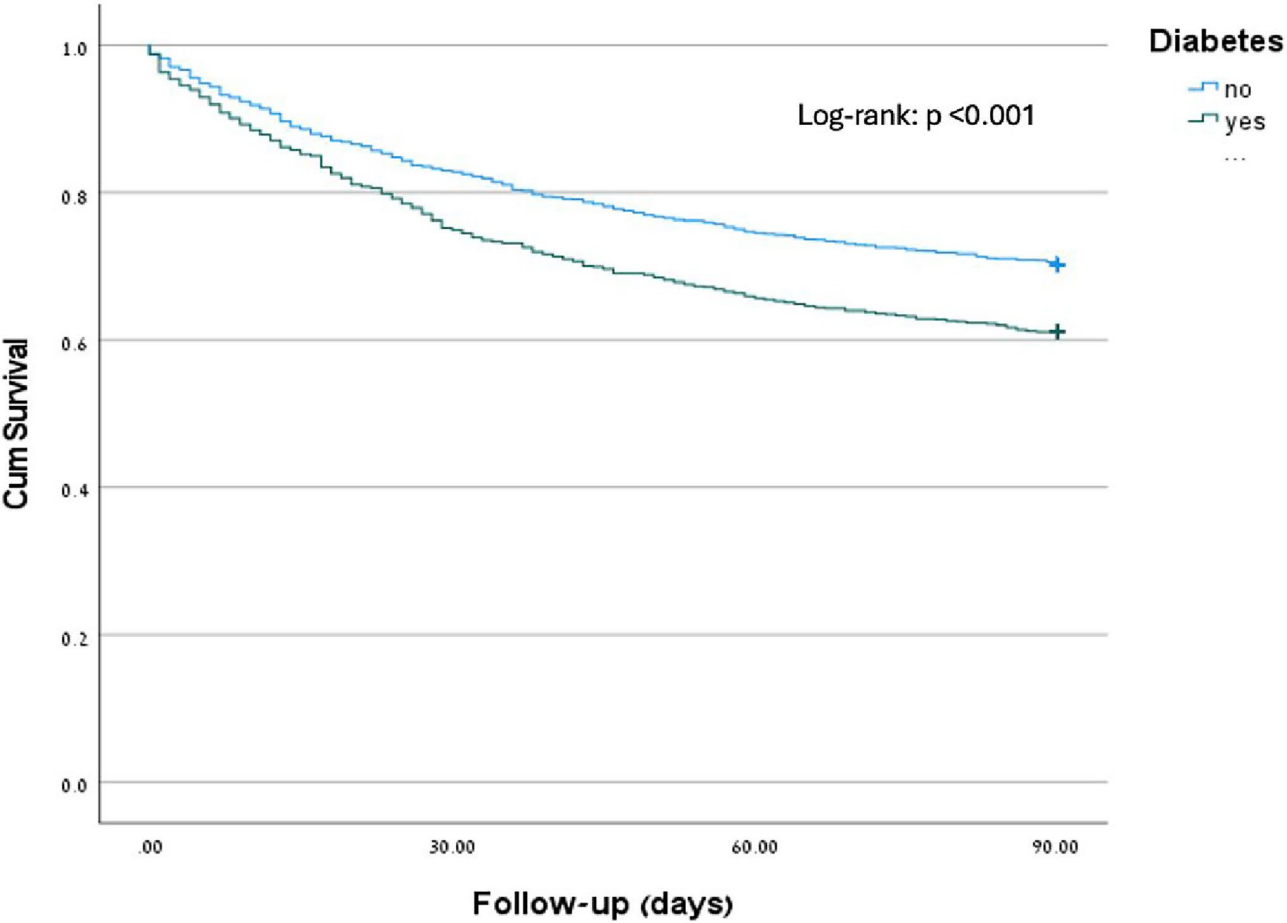
Kaplan–Meier diabetes mellitus and mortality within 90 days

Of the 2319 patients in our cohort, 782 (34%) died within 90 days of hospitalization: 391 (29.8%) of individuals without diabetes compared to 391 (38.9%) of those with diabetes (p < 0.001, log-rank test) (Fig. 1).

Table 3 summarizes the demographic and clinical characteristics of patients who died within 90 days after hospitalization compared to survivors. A significantly higher proportion of deceased patients had diabetes compared to survivors (50% vs. 39.9%, p < 0.001). Patients who died were significantly older (77.4 ± 13.2 vs. 67.9 ± 18.6 years, p < 0.001). Among those who died, there was a higher frequency of males (49.2% vs. 43.3%, p = 0.006) and individuals with low SES (27% vs. 25.1%, p = 0.028).

**Table 3:** Mortality within 90 days of hospitalization

Mortality variables	No N = 1537	Yes N = 782	P-value
Diabetes	614 (39.9)	391 (50.0)	< 0.001
Age	67.9 ± 18.6	77.4 ± 13.2	< 0.001
Male n (%)	665 (43.3)	385 (49.2)	0.006
SES n (%)			0.028
Low	386 (25.1)	211 (27.0)	
Middle	929 (60.4)	488 (62.4)	
High	215 (14.0)	77 (9.8)	
Missing	7 (0.5)	6 (0.8)	
Jews N (%)	1370 (89.1)	696 (89.0)	0.923
Hypertension N (%)	1011 (65.8)	637 (81.5)	< 0.001
Hyperlipidemia N (%)	1127 (73.3)	647 (82.7)	< 0.001
IHD n (%)	535 (34.8)	372 (47.6)	< 0.001
COPD n (%)	225 (14.6)	141 (18.0)	0.034
HF n (%)	345 (22.4)	301 (38.5)	< 0.001
CVA n (%)	386 (25.1)	274 (35.0)	< 0.001
CRF n (%)	527 (34.3)	382 (48.8)	< 0.001
Dialysis N (%)	107 (7.0)	40 (5.1)	0.084
Disability N (%)	433 (28.2)	380 (48.6)	< 0.001
Charlson score median	5.5 ± 3.2	7.1 ± 2.6	< 0.001
(IQR)	6 (3; 8)	7 (5; 9)	
WBC (n)	N = 1531	N = 780	< 0.001
Mean ± SD	11.2 ± 7.3	14.9 ± 12.6	
(IQR)	9.8 (6.8; 14.3)	12.4 (7.9; 18.5)	
Creatinine	N = 1530	N = 780	< 0.001
Median (IQR)	0.87 (0.63;1.37)	1.2 (0.71; 2.35)	
Albumin	N = 1490	N = 759	< 0.001
Mean ± SD	3.0 ± 0.60	2.5 ± 0.53	
Median (IQR)	3.0 (2.6–3.4)	2.5 (2.1–2.8)	

*SES* Socioeconomic status, *IHD* Ischemic heart disease, *HF* Heart failure, *COPD* Chronic obstructive pulmonary disease, *CVA* Cerebrovascular accident, *CRF* Chronic renal failure, *IQR* Interquartile range

Not surprisingly, comorbidities were more prevalent among patients who died within 90 days, including hypertension (81.5% vs. 65.8%), hyperlipidemia (82.7% vs. 73.3%), ischemic heart disease (47.6% vs. 34.8%), COPD (18.0% vs. 14.6%), heart failure (38.5% vs. 22.4%), cerebrovascular accidents (35.0% vs. 25.1%), chronic renal failure (48.8% vs. 34.3%), and disability (48.6% vs. 28.2%) (all p < 0.001). The Charlson Comorbidity Index (CCI) was also significantly higher in the deceased group (7.1 ± 2.6 vs. 5.5 ± 3.2, p < 0.001).

Regarding laboratory values, deceased patients exhibited higher white blood cell counts (13.8 ± 11.4 vs. 11.3 ± 7.4, p < 0.001), elevated creatinine levels (1.8 ± 1.6 vs. 1.5 ± 1.4, p < 0.001), and lower albumin levels (2.9 ± 0.67 vs. 3.4 ± 0.66, p < 0.001), indicating poorer overall health status.

### A multivariable analysis of factors associated with increased mortality

Cox regression analysis was performed to identify factors linked to the outcome of interest, such as mortality within 90 days. The variables included in the model were age, socioeconomic status (SES), ethnicity, comorbidities (e.g., COPD, hypertension, hyperlipidemia), and diabetes. The results are summarized in Table 4.

**Table 4:** Cox regression of factors associated with mortality within 90 days

variables	Hazard ratio	Confidence interval 95%	P-Value
Age	1.032	1.026–1.038	<.001
Gender (male)	0.777	0.674–0.896	<.001
SES			<.001
Low	0.857	0.704–1.042	0.123
High	0.627	0.472–0.834	0.001
Ethnicity	1.116	0.847–1.470	0.436
Hypertension	1.147	0.938–1.401	0.181
COPD	1.056	0.878–1.269	0.565
Hyperlipidemia	1.039	0.85–1.27	0.709
Diabetes	1.168	1.009–1.168	0.038

*SES* Socioeconomic status, *COPD* Chronic obstructive pulmonary disease

Diabetes was associated with a slightly increased risk of mortality, HR [1.168, 95% CI: 1009–1.353, p = 0.038], indicating that patients with DM had 16.8% higher odds compared to those without DM (p = 0.038). Age was also a strong predictor [HR = 1.03, 95% CI 1.02–1.04, p < 0.001]. SES showed a significant association; high SES was linked to a lower risk of mortality [HR = 0.63, 95% CI 0.47–0.63, p = 0.001]. Ethnicity was not significantly associated with the outcome (p = 0.436), and no significant associations were found for COPD (p = 0.565), hypertension (p = 0.172), or hyperlipidemia (p = 0.709).

## Discussion

The current study presents a comprehensive, relatively large, and unique platform for evaluating patients with CDI, focusing mainly on two important groups: those with diabetes mellitus (DM) and those without. It offers detailed insights into baseline characteristics, associated comorbidities, and factors related to clinical outcomes, including 90-day mortality among patients with CDI. Additionally, our study aims to assess diabetes as a potential predictor of increased CDI among hospitalized patients.

The baseline characteristics consistent with previously reported data have demonstrated an increased incidence of CDI among older patients with diabetes compared to the non-diabetic group (74.5 ± 12.0 vs. 68.6 ± 20.4) (p < 0.001). However, unlike earlier studies that noted female predominance among hospitalized diabetic patients with CDI, our current study reveals a significantly higher male predominance in DM-associated CDI compared to non-diabetic patients (47.9% vs. 43.3%, p = 0.029) [11, 13–15]

Limited data on SES and CDI exist. Safdar N et al. showed a significantly increased risk of hospital readmission among CDI patients residing in disadvantaged neighborhoods [16]. In line with previously reported data, the current study demonstrated a statistically significantly higher rate of CDI in patients with low SES. It is well known that host-related factors such as advanced age (≥ 65 years), severe underlying diseases, and decreased immune response effectiveness have long been recognized as major risk factors for adverse outcomes of infectious diseases, including CDI [17]. Additionally, T2DM can lead to an immunocompromised state, resulting in subsequent hospitalization and an increased risk of CDI [18]. Overall, the baseline characteristics mentioned above, namely males, older age, and low SES, defined a distinct clinical profile among diabetes patients diagnosed with CDI during hospitalization.

As expected, a higher burden of comorbidities among patients with diabetes and CDI was observed. This group of patients had significantly higher rates of hypertension, hyperlipidemia, ischemic heart disease, chronic renal failure, heart failure, and other conditions. This aligns with the well-established understanding that diabetes is often accompanied by a range of cardiovascular and metabolic disorders, which could potentially exacerbate the severity of CDI [11, 19]

The Charlson Comorbidity Index (CCI), a summary of comorbidity burden, was significantly higher in diabetic patients. Suggesting that diabetes is frequently associated with other severe comorbidities, further compounding the risks associated with CDI. A higher CCI score is known to correlate with increased morbidity and mortality in hospitalized patients, including those with infectious diseases like CDI [11].

Patients with DM showed significantly higher creatinine levels, a common complication of long-term diabetes. Additionally, albumin levels were somewhat lower in the CDI group with diabetes, indicating poorer outcomes for this population [20]. The white blood cell count (WBC) was significantly different between patients with and without diabetes (12.4 ± 9.6 vs. 12.1 ± 8.7, p = 0.098). Notably, in some instances of immunosuppression, such as those undergoing cancer chemotherapy or receiving hematopoietic and solid organ stem cell transplants, the WBC response may be excessively suppressed or even absent, making this severity marker difficult to interpret [21] [22].

In reviewing the literature, limited data were available for comparison of associated comorbidities, CCI, renal function tests, albumin, and WBC between patients with diabetes and those without [11]. This gap in the literature highlights the importance and uniqueness of our research. Overall, patients with DM and CDI exhibited significantly higher comorbidities, CCI, and creatinine levels, along with lower albumin levels.

While CDI was the primary diagnosis for all patients in this study, notable differences in coexisting conditions were observed between the two groups. Patients with diabetes exhibited a higher prevalence of cardiovascular diseases (10.8%), bloodstream infections (11.1%), and CDI itself (19.9%) as the principal diagnosis at admission. In comparison, non-diabetic patients experienced higher rates of gastrointestinal infections (8.5%), including CDI (24.8%). These data reinforce the idea that diabetic complications and immunocompromised status significantly affect patients with CDI. Contrary to our current findings, Ana López-de-Andrés et al. reported that the most common primary diagnoses for T2DM patients admitted with a secondary CDI were pulmonary disease or pneumonia (17.1%), heart disease (7.3%), and urinary tract infection (5.9%). Meanwhile, in non-diabetic patients, the most common diagnoses were pulmonary disease or pneumonia (15.3%), septicemia (5.9%), and urinary tract infection (4.5%)[11].

When examining 90-day mortality, patients with diabetes had a significantly higher mortality rate (38.8%) compared to those without diabetes (29.8%) (p < 0.001). This finding, consistent with previously reported data, highlights the increased vulnerability of diabetic patients to severe infections and suggests that diabetes may influence the course and severity of CDI [18, 23]. The current research revealed that diabetes, older age, low SES, and male gender were significant predictors of mortality. In contrast, higher SES was linked to a lower mortality rate.

The Cox regression analysis confirmed that diabetes is a significant predictor of 90-day mortality, with diabetic patients exhibiting moderately higher odds of dying within 90 days compared to non-diabetic patients (Exp(B) = 1.168, p = 0.038). Consistent with the current data, previously reported findings indicate that patients with diabetes are at greater risk for poorer outcomes following infections, including CDI [12]. Additionally, the identification of older age as a crucial predictor of mortality in our regression analysis (OR = 1.032, p < 0.001) suggests that for each additional year of age, the odds of mortality increase by 3.2%, particularly for patients with low SES as well. These predictors emphasize the importance of heightened vigilance and strict follow-up to reduce the mortality rate among hospitalized patients, especially those with diabetes. Notably, the Cox regression analysis model was limited to the above-reported variables, considering that other variables are part of diabetes complications, including ischemic heart disease, CHF, CVA, dialysis, and disability. Including these variables in the analysis, diabetes no longer shows statistical significance HR [1.062, 95% CI: 0.915–1.232, p = 0.429].

The following limitations of our study should be acknowledged. This is an observational retrospective study, and although we adjusted for several known confounding variables, the potential influence of unmeasured confounders cannot be ruled out. The analysis omits potentially confounding factors, such as antibiotic use and proton pump inhibitor exposure, which might introduce additional variability in interpreting the results.

## In conclusion

Patients with DM and CDI exhibited different comorbidities compared to those without diabetes. This study indicates that DM, older age, low SES, and male gender are linked to poorer outcomes, including mortality, among patients with CDI. Consequently, healthcare providers should take a more proactive approach in managing these patient groups.

## Data Availability

The datasets generated during and/or analysed during the current study are available from the corresponding author on reasonable request.

## Declarations

### Generative AI and AI-assisted technologies in the writing process

While preparing this work, the author(s) used Grammarly® to check for grammatical errors. After using this tool/service, the author(s) reviewed and edited the content as needed and take(s) full responsibility for the publication's content.
